# A Transformation-First Roadmap for Safe and Sustainable Emerging Advanced Materials

**DOI:** 10.1021/accountsmr.5c00370

**Published:** 2026-02-05

**Authors:** Swaroop Chakraborty

**Affiliations:** † School of Geography, Earth & Environmental Science, 1724 University of Birmingham, Edgbaston, Birmingham B15 2TT, U.K.; ‡ Centre for Environmental Research and Justice, 1724 University of Birmingham, Edgbaston, Birmingham, B15 2TT U.K.

## 1 Why SSbD Must Become a Default Design Constraint?

Advanced materials are being engineered at a pace that outstrips our collective ability to anticipate their life-cycle impacts. This mismatch is no longer academic. The 2025 Nobel Prize in Chemistry recognized the development of metal–organic frameworks (MOFs), signaling how modular, porous architectures have become enabling platforms for carbon capture, separations, sensing, and water technologies.^1^ In parallel, regulators and funders are shifting from “risk management after deployment” to prevention-oriented innovation, expecting credible SSbD evidence early in R&D, not retrofitted late.^2–4^

The European Commission’s Safe and Sustainable by Design (SSbD) Recommendation formalized this direction: chemicals and materials should be designed to provide a function while avoiding harmful properties, and sustainability should be ensured through a life-cycle perspective.^3^ then, the Joint Research Centre (JRC) framework has evolved through testing rounds (2023–2024) and has been updated again in 2025, reflecting a move toward clearer “SSbD fundamentals” and staged, decision-oriented assessment.^2,4^ Complementary work has crystallized best practice into roadmaps and operational guidance for advanced materials.^5,6^

For materials researchers, the implication is simple: functionality alone will not define success. If a material’s transformative trajectory^7,8^ (air → water/soil → biota → end-of-life) undermines performance or creates new hazards or functional failure, it will face regulatory friction, market rejection, or both. SSbD therefore cannot be an end-stage “tick box”; it must be treated as a design variable, optimized alongside the functional target.

This Viewpoint develops a practical SSbD playbook grounded in transformation science—an area where my work across engineered nanomaterial (ENM) dissolution, dopant-controlled release, bio/eco-corona formation, and the environmental transformation and ecotoxicology of MOFs. The central claim is that SSbD becomes actionable when we stop treating materials as static objects and instead treat them as trajectories. Three propositions recur across systems we and others study: (i) early commit-point chemistry can lock in downstream fate; (ii) abiotic stability does not imply biological inertness; and (iii) “safer” solutions are often achieved by controlling where, when, and in what form reactivity is expressed.

## 2 A Transformation-First SSbD Lens: Three Questions That Organize the Design Space

A tractable SSbD workflow starts with three questions that map directly onto design levers and measurement priorities.

**Q1. What is the first commit point?** In the first few moments after synthesis or deployment, hydration, ion speciation, surface reconstruction, and corona adsorption can create a new “material identity” that persists even if bulk structure appears unchanged. Commit points matter because later environments act on the already-transformed interface.^9^

**Q2. What is released (and when)?** For many advanced materials, hazard and performance drift are driven less by the pristine solid than by dissolved ions, ligands, additives, or secondary phases produced during aging or in vivo processing. Release kinetics- rather than single end-points- often provide the most informative design feedback.

**Q3. Can the material be retrieved, reused, or benignly disappear?** Circularity and exposure prevention are frequently achieved by immobilization, modular disassembly, regeneration strategies, or designing for benign end-of-life transformations. A material that is safe in a beaker but cannot be contained, retrieved, or recycled in a product is rarely SSbD-compatible.

These questions can be asked of almost any platform, but they are especially urgent for high-surface-area systems (porous frameworks, nanohybrids, 2D materials) where interfacial chemistry dominates behavior and where the gap between “as-synthesised” and “as-deployed” identity is typically largest.

## 3 Four SSbD Design Levers, Illustrated with Breakthrough Exemplars

### 3.1 Control Dissolution, Speciation and Secondary Phases: Release-Centered SSbD

Dissolution and ion release are among the most powerful predictors of both function loss and toxicity for metal-containing ENMs.^10,11^ One critical materials design consideration is dissolution – the release of ions or degradation products from a material (Figure 1, Step 2). Dissolution often governs a nanomaterial’s fate and bioavailability. For instance, soluble nanoparticles may release toxic ions, whereas insoluble particles might persist and accumulate. Over a decade ago, dissolution was identified as a key factor in nanotoxicology, and since then our understanding of nanomaterial surface chemistry and transformations has deepened.^10^ Today dissolution is recognized as a tunable property that can be leveraged for safer design. For example, zinc oxide (ZnO) nanoparticles gradually dissolve and release Zn^2+^ ions – these ions can bind to and inhibit critical enzymes like acetylcholinesterase, potentially leading to neurotoxic effects in organisms. Such findings underscore that uncontrolled dissolution can be a hazard.^12^ An SSbD strategy, then, is to deliberately adjust the composition or structure of nanomaterials to modulate their dissolution rates.

**Figure 1:**
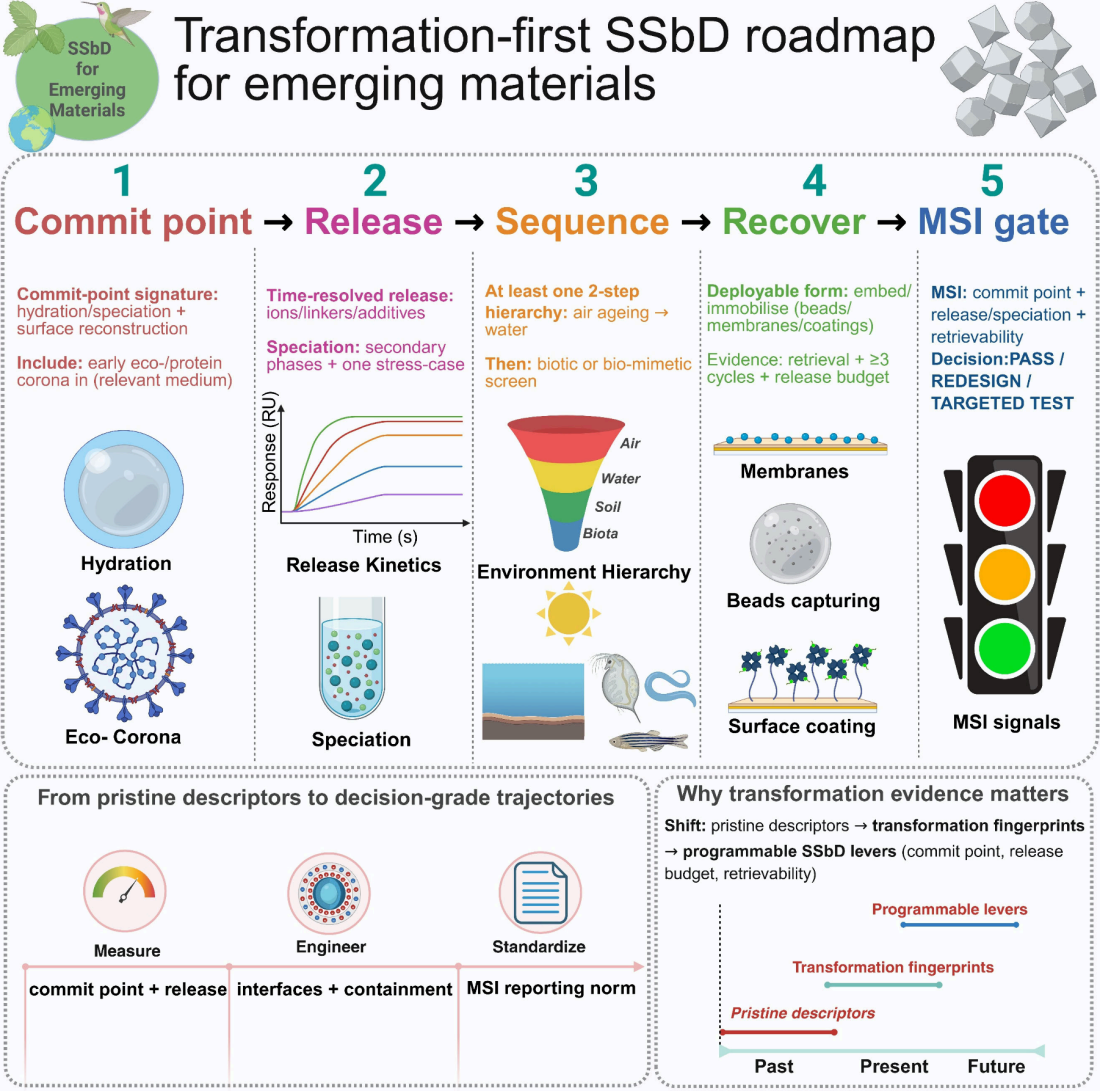
Transformation-first SSbD roadmap for emerging materials. The Viewpoint is mapped into five linked steps: commit-point signature, time-resolved release/speciation, sequence testing to generate trajectory maps, recoverability via deployable forms with ≥ 3 regeneration cycles and a release budget, and an MSI decision gate (PASS/REDESIGN/TARGETED TEST). Lower panels summarize the shift to decision-grade trajectories. (2026) Created in BioRender. Chakraborty, S. (2026) https://BioRender.com/ysqt2ti.

A landmark safe-by-design demonstration showed that Fe-doping of CuO nanoparticles suppresses Cu dissolution and substantially reduces cytotoxicity compared with undoped CuO, while retaining functional utility.^13^ Mechanistically, compositional tuning altered surface chemistry and promoted more stable phases, lowering bioavailable Cu. The broader lesson is not that doping is always safer, but that compositional tuning can steer dissolution/speciation and thereby help decouple performance from hazard. My own work argues that SSbD needs to treat “release” as a hypothesis to be tested across relevant environments rather than as a property inferred from pristine characterization. In a study on doped ferrite nanoparticles,^14^ we explicitly separated particulate-driven effects from ion-driven effects across media and showed that doping can shift dissolution pathways and therefore the balance between particulate versus ionic bioactivity. This matters because the same dopant that improves a functional metric (magnetic response, catalytic rate, optical absorption) may also redirect dissolution to a more bioavailable or more persistent route, creating an unexpected trade-off. Similarly, in a 2021 study by Guo et al. silver and other metallic nanomaterials were “aged” (biotransformed) in simulated biological fluids and then tested against a human blood–brain barrier model; the bio transformed particles showed reduced penetration compared to pristine particles. This suggests that early stage transformations can detoxify nanomaterials, and by harnessing this knowledge, we might intentionally pretreat or formulate nanomaterials so that by the time they contact sensitive tissues, they are already in a safer, less reactive form.^15^ For MOFs, release-centered SSbD is equally important.^16,17^ Framework “stability” is often reported as retention of crystallinity or surface area, yet small releases of node metals or linkers can dominate exposure when the functional context is water treatment, food, antimicrobial coatings, or consumer contact.^18,19^ A release-centered approach therefore asks: what is the maximum tolerable leaching under realistic ionic strength and organic matter? Do oxidants, chelators, or pH swings trigger release spikes? Does release correlate with function loss or with a change in biological response? Addressing these questions early enables rational design (node selection, mixed-metal strategies, linker engineering, surface passivation) rather than empirical trial-and-error.

#### Design Implications

Treat release as a tunable property with explicit targets (e.g., “<1% metal leaching over 7 days in defined media”; “no acute ion-spike within first 2 h”; or “triggered dissolution into benign species at end-of-life”). Publish release kinetics, not only end-points; and report a minimal set of conditions that bracket plausible “best-case” and “stress-case” scenarios (ionic strength, pH, natural organic matter, oxidants).

### 3.2 Design for Exposure Sequence and *In Vivo* Transformation: Trajectory-Centered SSbD

A persistent assumption in water-sector materials is that abiotic stability predicts ecological safety. We recently overturned this assumption for nanoscale UiO-66. UiO-66 is a Zr-based MOF widely regarded as exceptionally robust, tracing back to the original Zr_6_ “inorganic building brick” work that established its high stability.^20^ UiO-66 remained structurally intact after extended abiotic aging in natural borehole water yet underwent rapid *in vivo* transformation inside freshwater crustaceans *Daphnia magna*, producing chronic reproductive toxicity.^20^ The key point is not that UiO-66 is “unsafe”, but that the usual screening proxy-abiotic stability- failed to predict biological processing routes. The practical consequence is that “single-medium stability” claims can be misleading for porous and reactive systems. Biological processing can introduce transformation routes (gut chemistry, enzymatic environments, complexation, local pH gradients, redox microenvironments) that are invisible to abiotic stability tests. Trajectory-centered SSbD therefore requires sequential exposure testing that matches plausible environmental hierarchies (e.g., oxidative air aging → natural water → biota), combined with end points that can reveal long-latency effects where short-term mortality is absent (Figure 1, Step 3).^7^ Importantly, this does not mean every project must run full chronic tests from day one. Rather, early screening should be designed to reveal commit points and likely failure windows. For example: does early exposure to natural organic matter or proteins alter aggregation and uptake? Does *in vivo* processing generate new secondary phases or release spikes? Does an apparently “stable” framework fragment or amorphize in ways that change bioavailability? Answering even a subset of these questions early can direct design choices: particle size and morphology; linkers and defect control; polymer encapsulation; or deployment as a recoverable composite rather than a free powder.

#### Design Implications

Replace “stable in water” claims with sequence-aware transformation maps. For porous materials intended for water-sector deployment, incorporate at least one biotic or biomimetic transformation screen that can detect *in vivo* processing routes (for example a gut-simulant, enzyme-rich medium, or a small-organism model such as *Daphnia magna*, zebrafish, *C. elegans*) (Figure 1, Step 1 and Step 3).

### 3.3 Engineer Recoverability and Circularity by Immobilization and Modularity: Systems-Centered SSbD

Many advanced materials fail SSbD not because they are intrinsically hazardous, but because they are difficult to contain, retrieve, or recycle (Figure 1, Step 4). Many nanomaterials owe their reactivity (and functionality) to high surface area and chemical activity—traits that also increase their propensity to leak or cause harm. Encapsulating or supporting such materials on more inert substrates can mitigate these downsides without losing performance. For instance, our group developed polymer-embedded MOF beads as a safe, sustainable platform for water purification.^17^ The composite material consists of a nanoscale copper-based MOF (BNMG-1) encapsulated in a biodegradable cellulose acetate (CA) polymer matrix. By itself, the nano-MOF is extremely effective at binding heavy metals, but if used in isolation it could present stability issues (the MOF contains Cu^2+^ and might leach) and would be difficult to recover from water. Embedding the MOF in CA solved these issues: the CA–MOF beads achieved over 80% removal of Pb^2+^ from complex aqueous solutions (including real canal water and artificial seawater), even in the presence of competing ions and organic matter. Compared to the bare MOF, the composite beads showed dramatically enhanced selectivity for Pb^2+^—for example, the separation factor for Pb^2+^/Ni^2+^ improved from ∼57 (bare MOF) to ∼221 with the CA–MOF beads. Importantly, copper leaching from the MOF was reduced to <5% (and <1% in many conditions), since the polymer matrix retains the MOF particles and likely sequesters any dissociated metal. The beads could be easily filtered out after use and reused, maintaining >95% of their Pb removal efficacy over at least three cycles. This work explicitly “aligns with the safe and sustainable by design (SSbD) framework”—it demonstrates an eco-friendly, scalable solution where a high-performance material (a nanoMOF) is made safer by integrating it into a stable, recoverable form. In related work, MOF bead architectures have been extended toward selective rare-earth capture and recovery, supporting circular-economy targets for critical materials.^21,22^ This “materials-in-a-device” framing matters because the exposure unit in real deployments is often a product or composite, not a pristine nanopowder. Designing recoverability (shape-stable beads, cartridges, membranes, magnetic separation) can simultaneously reduce exposure potential and improve adoption by end users. It also allows SSbD to be engineered at the systems level: a slightly less active powder can be preferable if it can be deployed in a cartridge that is regenerated and used, preventing downstream release and lowering whole-life impacts.

#### Design Implications

For any nanostructured active intended for water or consumer contact, demonstrate (i) how the material is retrieved in use, (ii) how it is regenerated or replaced, and (iii) how active-component release behaves under realistic use and regeneration cycles. Treat containment and retrievability as a design objective, not an implementation detail.

### 3.4 Program the Interface: Eco-/Protein Corona as a Design Variable (Identity-Centered SSbD)

A foundational breakthrough in nanoscience is the recognition that particles acquire a “biological identity” through biomolecular coronas that dictate uptake, trafficking and toxicity.^23^ For SSbD, the corona should be viewed less as a nuisance and more as a programmable layer. Surface engineering can mitigate corona-driven loss of intended interactions; for example, Polyethylene Glycol “backfilling” was shown to reduce corona formation and recover specific targeting in protein-rich environments.^24^ In environmental systems, eco-coronas formed from natural organic matter, proteins and metabolites can similarly reshape transport and uptake, sometimes attenuating toxicity and sometimes amplifying it by stabilizing dispersions or facilitating uptake.^23^

Hybridization can also involve combining two nanomaterials so that one moderates the risks of the other. A case in point is the design of graphene oxide–gold (GO–Au) nanohybrids for safer biomedical use. Pristine graphene oxide sheets tend to aggregate in biological media and can induce oxidative stress in cells, whereas gold nanoparticles (AuNPs) are more inert but need a support to prevent agglomeration. In an SSbD-minded study, Ibrahim et al. (2025) attached tiny AuNPs onto GO sheets.^25^ The presence of gold markedly increased the dispersion stability of GO in aqueous environments—the GO-Au hybrids retained over 98% of their initial absorbance in water and over 95% in cell culture medium after 48 h, whereas GO alone showed significant sedimentation. The gold decoration also appeared to “passivate” GO’s surface reactivity. Toxicity assays in zebrafish embryo cells showed a clear difference: at 150 μg/mL, plain GO caused cell viability to drop to ∼39%, while GO–Au at the same dose was notably less toxic (54.5% viability). GO generated roughly twice as many reactive oxygen species (ROS) in cells compared to the GO–Au hybrid and GO induced significantly higher cell death rates (e.g., necrosis in 53% of cells at 100 μg/mL, vs only ∼15% with GO–Au). These findings demonstrate that incorporating AuNPs reduced GO’s cytotoxicity and oxidative stress by enhancing colloidal stability and perhaps altering surface interactions. In other words, joining materials can mitigate hazard: the GO–Au composite had better behavior in biological systems yet still retained the combined functionalities of conductive graphene and plasmonic gold.

Likewise, in the antimicrobial realm, surface engineering can be employed for safety. Silver nanoparticles are potent antimicrobials but continuous release of Ag^+^ ions is undesirable for health and the environment. Malvi et al. (2024) tackled this by depositing silver as discontinuous nanoislands onto stainless steel—effectively creating a nanopatterned coating rather than a uniform film.^26^ The “nano-island” design dramatically reduced total silver usage (over 90% less Ag than a fully coated film, since most of the steel surface remained uncoated) while still providing excellent antimicrobial and even antiviral performance. By varying the Ag thickness from ∼3 nm to ∼45 nm, they achieved a transition from isolated islands to a nearly continuous layer, which affected the coating’s properties. Discrete nanoislands reduced surface hydrophilicity by ∼57% and increased surface roughness by ∼50%, which actually enabled a controlled release of Ag ions and nanoparticles. This controlled slow release provided potent germ-killing effects with minimal silver leaching, eliminating the need for full Ag coverage. Stability tests confirmed that the nanoislands remained intact and retained antimicrobial efficacy over time—even after >10 wash and autoclave cycles, the coatings showed no significant loss of activity. This SSbD nanoisland approach offers a cost-effective, scalable solution for antimicrobial surfaces, achieving substantial microbial and viral inhibition while minimizing silver use and leaching. It essentially uses just enough nanomaterial to get the job done and immobilizes it in a way that prevents excessive release.

#### Design Implications

Characterize corona formation and its functional consequences in at least one relevant complex medium (environmental and/or biological). Use surface chemistry to stabilize a benign corona or prevent hazardous adsorption, and report how corona formation shifts uptake, release, aggregation and biological response.

### 3.5 Make Commit Points Measurable: The SSbD Toolbox Has Matured

Transformation-first SSbD only works if we can “measure” early time changes with enough chemical specificity to guide redesign. The good news is that the analytical toolbox is now rich enough that “mechanism-informed SSbD” is practical for many groups.

#### Speciation-Aware Analytics

Element-specific methods (e.g., X-ray absorption spectroscopy) can distinguish oxidation state and coordination changes that are invisible to bulk crystallinity or surface-area metrics, and can pinpoint when a node, dopant, or secondary phase becomes bioavailable.^27^ When coupled to dissolution measurements (Inductively Coupled Plasma -Mass Spectrometry) and particle sizing (using Dynamic Light Scattering/Electron Microscopy), these approaches allow a direct link between “what changes” and “what is released”.

#### Time-Resolved Commit-Point Capture

Many decisive transformations occur rapidly (in seconds or minutes), not days. Microfluidic and rapid-mixing strategies (including stopped-flow designs) make it possible to sample the first seconds-to-minutes of hydration, ion exchange, and corona adsorption-exactly the window in which later behavior is often “locked in”. Even if full operando setups are not available, disciplined early time sampling (e.g., milliseconds-minutes time points) is often sufficient to reveal release spikes and early aggregation that predict later outcomes.^9^

#### Biointerface Characterization

Proteomics-enabled corona profiling and environmentally relevant conditioning media (eco-corona) now allow interface engineering to be treated as a controllable variable rather than an uncontrolled artifact. A practical SSbD standard is to report at least one complex-medium interaction experiment that links corona formation to a functional or biological end point (uptake, aggregation, release, oxidative stress).^23^

#### Decision Support and Tool Integration

OECD-led SSbD initiatives increasingly provide inventories of tools, platforms, and integrative systems that help innovators select “right-sized” methods at early stages and expand only when red flags appear. This is aligned with the staged logic of the JRC framework: do not demand perfect data on day one; demand design-informative data at each decision gate.^2,3,5^

The central point is that the field no longer lacks methods; it lacks shared expectations about which measurements are “minimum” and which are “contingent”. That is a cultural problem, not a technical one.

## 4 Roadblocks and Near-Term Priorities

SSbD discussions often fail because they either (a) stay at the level of slogans, or (b) demand a life-cycle completeness that is unrealistic for early stage research. In my view, there are five roadblocks that are both common and solvable—especially for the materials community.

### Roadblock 1

Pristine-only characterization. A large fraction of the literature still treats “as-synthesised” structure as the relevant identity. Yet commit-point transformations (hydration, ion exchange, surface reconstruction, corona adsorption) can dominate later behavior. **Priority:** move to “as-deployed” characterization, even if minimal.

### Roadblock 2

Unreported release and speciation. Many papers report performance (e.g., adsorption capacity) without quantifying what leaches, when it leaches, and in what chemical form. **Priority:** routine time-resolved release reporting and, when possible, speciation-aware methods that distinguish particulate from ionic contributions.^13,14^

### Roadblock 3

Mismatch between test media and use context. “Stability in Deionized water” or “toxicity in one cell line” is often not informative for a water-sector cartridge, a medical device, or a surface coating. **Priority:** match at least one screening medium and one hazard screen to the intended context (natural water, serum-containing medium, conditioning media, relevant organism model) and be transparent about what is and is not represented.

### Roadblock 4

No retrieval story. If a material cannot be contained, separated, regenerated, or disposed of benignly, its SSbD ceiling is low regardless of performance. Priority: demonstrate a realistic deployment form (bead, membrane, coating, cartridge) and quantify release across cycles.^17,21,26^

### Roadblock 5

Weak redesign loops. SSbD is an iterative process, yet research often present safety as a one-off assay at the end. **Priority:** articulate a redesign loop explicitly: “observation → mechanism → design change → re-test” and make trade-offs transparent. Framework and roadmap papers emphasize that SSbD succeeds when it is operationalised as staged decision-making rather than as a final certificate.

These priorities do not require every laboratory to become a full Life Cycle Analysis or regulatory science unit. They require a shared norm that “safe and sustainable” claims must be supported by at least (i) a commit-point/trajectory perspective, (ii) release quantification, and (iii) a containment/retrieval argument.

## 5 Operationalising SSbD in Materials Research: A “Minimum SSbD Information” Checklist

The most common failure mode I see in SSbD discussions is overambition: attempting to solve every life-cycle question at once. The JRC framework is valuable precisely because it structures SSbD as an iterative decision process with staged information needs. For early stage materials papers, having a pragmatic “minimum SSbD information” (MSI) checklist that fits typical materials programmes while still shifting the field away from pristine-only claims (Figure 1, Step 5).

(1) **Functional unit and use context.** Define the service provided (e.g., milligram contaminant removed per gram material per cycle; catalytic turnovers per mass of active; antimicrobial log reduction per surface area) and identify plausible exposure routes during synthesis, use and end-of-life. This anchors sustainability metrics and hazard testing to a meaningful basis.
(2) **Commit-point screen.** Report the first-hours transformation signature in one relevant complex medium (a natural water with ionic strength and organic matter; serum-containing medium; or an application-relevant conditioning medium), including simple readouts such as aggregation behavior (e.g., sedimentation), surface charge, and early release.
(3) **Release kinetics**. Quantify metal/ligand/additive release over time (not just a single time point). Include at least one accelerated-stress condition that reveals worst-case leaching (oxidants, pH extremes, chelators), because stress tests are often where design-relevant differences become apparent.
(4) **Recoverability and regeneration**. Demonstrate how the material is retrieved (separation approach) and whether performance and release remain acceptable over repeated cycles.
(5) **Hazard flagging with fit-for-purpose assays**. Use a small panel of mechanistically informative assays matched to the use context. For water-sector materials, an aquatic model and chronic-relevant end points may be necessary even when acute lethality is absent. For reactive nanohybrids, oxidative stress and membrane integrity are often more design-informative than a single viability readout. Safe-by-design frameworks for nanomaterials emphasize iterative testing and redesign; the key is to ensure assays inform specific design changes rather than functioning as “after-the-fact” reporting.^28,29^

This MSI checklist aligns with the increasing availability of SSbD toolkits and decision-support approaches from the OECD and others, which are designed to support early stage innovation when data are limited. The culture shift required is modest but important: we should expect papers to report “what changes first”, “what is released”, and “how is it contained”, not only “what is it when pristine”.

## 6 Outlook

SSbD becomes actionable when materials are treated as trajectories with measurable early commit points-rather than as pristine, static substances. SSbD is often framed as an additional burden; I view it as an enabling constraint that accelerates translation by reducing late-stage surprises. As summarized in Figure 1, the field is shifting from pristine descriptors to decision-grade trajectories, where transformation fingerprints enable programmable SSbD levers. The 2025 Nobel recognition of MOFs captures a broader truth: advanced materials are now central to climate, water and health solutions, and therefore they will be scrutinized as dynamic systems that interact with the environment. A transformation-first SSbD playbook-commit points, release control, interface programming and recoverability-turns that scrutiny into an engineering problem the community is well-equipped to solve. If SSbD is to become routine, journals and reviewers must also evolve expectations. The minimum information requirements proposed above are intentionally lightweight; they are designed to be feasible within a typical materials program while still providing a defensible basis for “safe and sustainable” claims. The reward is clear: materials that are not only performing higher on day one, but also remain functional, containable and acceptable across their life cycle.
